# Hyperferritinemia Is Associated with Insulin Resistance and Fatty Liver in Patients without Iron Overload

**DOI:** 10.1371/journal.pone.0003547

**Published:** 2008-10-28

**Authors:** Robert Brudevold, Torstein Hole, Jens Hammerstrøm

**Affiliations:** 1 Department of Medicine, Aalesund Hospital, Aalesund, Norway; 2 Department of Medicine, St. Olavs Hospital, Trondheim, Norway; 3 Norwegian University of Science and Technology (NTNU), Trondheim, Norway; MetroHealth Medical Center, United States of America

## Abstract

**Objective:**

During the last 10 years we have experienced an increasing number of referrals due to hyperferritinemia. This is probably due to increased awareness of hereditary hemochromatosis, and the availability of a genetic test for this condition. Most of these referred patients were over-weight middle-aged men with elevated ferritin levels, but without the hemochromatosis-predisposing gene mutations. We evaluated the relationship between hyperferritinemia and the metabolic syndrome in 40 patients.

**Methods:**

Forty consecutive patients referred for hyperferritinemia were investigated. The examination programme included medical history, clinical investigation and venous blood samples drawn after an overnight fast. This resulted in 34 patients with unexplained hyperferritinemia, which were further examined. Liver biopsy was successfully performed in 29 subjects. Liver iron stores were assessed morphologically, and by quantitative phlebotomy in 16 patients.

**Results:**

The majority of the patients had markers of the metabolic syndrome, and 18 patients (52%) fulfilled the IDF-criteria for the metabolic syndrome. Mean body mass index was elevated (28,8±4,2), mean diastolic blood pressure was 88,5±10,5 mmHg, and mean fasting insulin C-peptide 1498±539 pmol/l. Liver histology showed steatosis and nuclear glycogen inclusions in most patients (19 out of 29). Only four patients had increased iron stores by histology, of which two could be explained by alcohol consumption. Fourteen of 16 patients normalized ferritin levels after phlebotomy of a cumulative blood amount corresponding to normal iron stores. Ferritin levels were significantly related to insulin C-peptide level (p<0.002) and age (p<0.002).

**Conclusion:**

The present results suggest that liver steatosis and insulin resistance but not increased iron load is frequently seen in patients referred for suspected hemochromatosis on the basis of hyperferritinemia. The ferritin level seems to be positively associated to insulin resistance.

## Introduction

Hemochromatosis is a disease with vague symptoms, serious complications and an effective treatment. Since the genetic test for hereditary hemochromatosis became available in 1996, focus on the disease has increased [1]. Serum-ferritin is frequently used as a first line blood test when iron overload is suspected. However, ferritin is also elevated in inflammatory states and malignant diseases [2].

The hemochromatosis-associated genotype (homozygous C282Y mutation of the *hfe-* gene) is found in about 85% of patients with hereditary hemochromatosis [3]. The *hfe*-gene codes for the HFE-protein, which plays an important role in iron absorption in enterocytes [4].

A possible link between hepatic iron-overload and insulin resistance has been reported [5]–[7], and an association of non-alcoholic steatohepatitis (NASH) and insulin resistance has also been shown [8]. In some patients with NASH ferritin was elevated.

Since 1996 we have experienced an increasing number of referrals from general practitioners of patients with hyperferritinemia. Many patients were overweight, middle-aged men with vague symptoms such as tiredness, fatigue, myalgia and arthralgia. Liver transaminases, especially alanine amino transferase (ALT), were often elevated. Transferrin saturation was within the reference range and the HFE gene mutation was negative. Liver biopsy often showed steatosis and nuclear glycogen inclusions, which is a frequent finding in prediabetes and diabetes mellitus type 2 (DM2) [9]. Usually there were no definite signs of iron overload or other pathology.

We have examined the relationship between hyperferritinemia, iron-load and insulin resistance in patients referred for elevated ferritin levels and suspicion of hemochromatosis. Patients were examined and treated according to best practice.

## Methods

### Patients

During 2001 40 patients were referred to the hematology clinic at Aalesund Hospital due to elevated ferritin levels and suspicion of hemochromatosis. This is an observational study with retrospective analysis of the results of the examination programme. All patients gave oral consent to the examination programme. Due to the retrospective observational nature of the study, the Regional ethics committee of Mid-Norway had no comment to the study, and as the examinations themselves were part of a clinical quality assurance program, they did not need formal approval from the ethics committee. However, all patients gave oral consent to the use of clinical data for research purposes.

Five patients had hereditary hemochromatosis and one had hemosiderosis secondary to excessive iron intake. Hence 34 patients (33males, 1 female) had no *hfe*-mutation known to predispose for hemochromatosis (neither homozygous for C282Y nor compound heterozygous, i.e. heterozygous for both C282Y and H63D) and was further examined to reveal other causes for hyperferritinemia.

### Examinations

A structured examination programme was performed including medical history, clinical examination, laboratory tests and liver biopsy. Reason for taking liver biopsy was hyperferritinemia/elevated liver transaminases where examinations so far had not given any explanation for the deviations. Examinations did not deviate from what is usually recommended for follow-up of such patients. The HUNT-study [10] was used as reference population regarding BMI (women; 26.4 kg/m^2^, men; 26.5 kg/m^2^), waist-hip ratio (women; 0.77±0.06, men; 0.89±0.06), systolic blood pressure (women; 136.8 mmHg, men; 140.1 mmHg) and diastolic blood pressure (women; 78.8 mmHg, men; 81.9 mmHg).

### Laboratory tests

Venous blood samples were drawn after overnight fast.

S-iron (reference values, women; 11–23 µmol/l, men; 14–29 µmol) was analysed using Colorimetric assay (CFAS-Roche, Mannheim, Germany). Serum-glucose (reference value 3.6–5.8 mmol/l), aspartate amino tranferase (AST) (reference values, women; <35 U/l, men; <50 U/l), alanine amino transferase (ALT) (reference values, women; <35 U/l, men; <50 U) were measured by UV-test (CFAS-Roche, Mannheim, Germany). C-reactive protein (CRP) was measured by a turbidimetric method (CFAS-Roche, reference value <6 mg/l). Hemoglobin A1C (HbA1C) (reference value, 3.5–6.5%) used ion-change-chromatography (HPLC-Biorad Variant Hb testing system, Marnes-La-Cocuette, France). Ferritin (reference values, women; 10–250 µg/l, men; 20–250 µg/l) and total iron binding capacity (TIBC)(reference value 46–80 µmol/l) were analyzed by nephelometry (Dade Behring. Marburg, Germany). Transferrin saturation (%) = s-iron/TIBCx100 (reference value, 15–45%). Fasting insulin C-peptide (reference value 240–720 pmol/l) was analysed by chemiluminescence method (DPC-immulite. Liamberis, Gwynodd, LL55 4EL, UK). HFE mutation was analyzed measuring fragment-length polymorphisms by PCR [1].

*Liver biopsies* were ultrasound-guided with 18-Gauge needles. Iron content was evaluated according to Rowe [11]: Grade 0–1 is within normal limits, and grades 2+ to 4+ represent distinct iron increase (70–850 µmol/g dry weight).

### Phlebotomy

Iron stores were also estimated by quantitative phlebotomy in 16 patients. One gram of hemoglobin contains 3.4 mg iron, and the amount of iron removed by phlebotomy can thus be calculated [12]–[13]. The normal amount of storage iron in males, measured by quantitative phlebotomy, has been shown to be about 750 mg [14]–[15].

### Definitions

There have been several definitions of the metabolic syndrome (WHO 1999/NCEP 2001)[16]–[17]. We used the recent simplified definition recommended by the International Diabetes Federation (IDF), which emphasizes waist circumference (women; >80 cm, men; >94 cm) in addition to minimum 2 of the following criteria: HDL-cholesterol <1.03 mmol/l (men), blood pressure >130/85 mmHg, triglycerides >1.7 mmol/l, or fasting blood glucose >5.6 mmol/l [18].

### Statistical analyses

Normal distribution of data was verified by normality plots. All normally distributed measurements are presented as mean and standard deviations (SD), otherwise median and range were used. Comparisons of normal distributed parameters were done by t-tests. Univariate and multiple forward linear regression analyses were used to investigate relations between s-ferritin and other variables. Logaritmic transformation of s-transferrin was used to obtain normal distribution. All tests were 2-sided, and a p-value <0.05 was considered significant. No adjustments for multiple testing were performed. All analyses were performed with SPSS 13.0 (SPSS Inc, USA).

### Role of funding source

The funding source had no involvment in the conduction of the study.

## Results

The mean age was 53 (12.6) years.

Twenty-three percent reported DM2 among relatives, and 23% also reported some kind of iron-overload-related problem in the family. Fourteen percent knew about relatives with some kind of malignant liver disease (table 1).

**Table 1:** Demographic and clinical characteristics of the study population (n = 34).

Parameters	Number (%)
Male	33 (97)
Age (mean/range)	53/28–77
Hypertension	12 (35)
Type 2 diabetes	4 (12)
Gout	3 (9)
Coronary artery disease	2 (6)
Hyperlipidemia	2 (6)
Smokers	6 (17)
No alcohol consumption	12 (35)
Minimal alcohol consumption	20 (59)
Moderate alcohol consumption (<60 g/week)	2 (6)
BMI>30	16 (47)

Symptoms reported by the patients were vague. Fifty-eight percent reported fatigue and 17% arthralgia/myalgia.

Seventeen patients (50%) were without any medication. Most used medicines were betablockers (7 patients), calcium-channel blockers (5 patients), ACE-inhibitors (4 patients) and metformin (4 patients).

TSH, CRP, Sedimentation rate (SR), s-electrophoresis and transferrin saturation were all within reference limits (Table 2).

**Table 2:** Clinical and biochemical characteristics (n = 34).

Parameter	Reference values	Mean	Standard deviation	Range
Body mass index	26.5 (men)	28.8	4.2	23–38
Waist/hip ratio	0.89 (men)	0.97	0.07	0.83–1.17
Systolic blood pressure(mmHg)	140.1 (men)	144	19	120–195
Diastolic blood pressure (mmHg)	81.9 (men)	88.5	10.5	64–114
Heart rate (beats/min)		75	13	50–116
Transferrin saturation (%)	15–45	39	10.2	12–57
Ferritin (µg/l)	20–250	665.5	369.4	283–2190
ALT (U/l)	<50 (men)	75	44.2	22–207
Triglycerides (mmol/l)	0.6–2.2	1.8	1.3	0.5–6.5
LDL-cholesterol (mmol/l)	1.7–5.4 (men)	3.8	1.1	1.2–6.1
HDL-cholesterol (mmol/l)	0.80–2.00 (men)	1.3	0.3	0.72–2.21
HbA1C (%)	3.5–6.5	5.8	1.26	4.5–9.2
Insulin C-peptide (pmol/l)	240–720	1498	539	503–2988
Fasting glucose (mmol/l)	3.6–5.8	6.4	2.29	4.3–14.7

ALT = alanine amino transferase.

Mean values for body mass index, waist circumference, waist/hip ratio and diastolic blood pressure were above cutoff values set by IDF regarding criteria for the metabolic syndrome.

Thirty-two out of 34 patients (94%) had elevated insulin C-peptide indicating hyperinsulinemia/insulin resistance.

Mean glucose was slightly elevated (6.41±2.2 mmol/l), s-triglyceride was in upper normal range, mean HDL-cholesterol was in lower normal range, and mean ALT was elevated.

*Hfe-* mutation analyses showed that 22 patients were heterozygous for C282Y, and twelve had neither C282Y nor H63D. There were no compound heterozygotes.

None of the present genetic combinations are known to predispose for iron overload, although heterozygous C282Y has been associated with elevated serum iron markers [19].

Liver histology is reported in table 3.

**Table 3:** Liver histology (n = 29).

Histologic findings	Number (%)
Normal	1 (3)
Nuclear glycogen inclusions	17 (59)
Steatosis	19 (66)
Cirrhosis (micronodular)	1 (3)
Non alcoholic steatohepatitis	1 (3)
Alcoholic steatohepatitis	2 (6)
Hemosiderin grade* 0	15 (51)
Hemosiderin grade 1	10 (34)
Hemosiderin grade 2	3 (10)
Hemosiderin grade 3	1 (3)
Hemosiderin grade 4	0 (0)

* Grading according to reference 11.

The majority of patients had liver steatosis and nuclear glycogen inclusions. Hemosiderin was within normal limits in 86% of the patients. The one patient with hemosiderin content grade 3 was diagnosed with steatohepatitis and surplus alcohol consumption (carbohydrate deficient transferrin>5%). The other patient with alcoholic steatohepatitis had also cirrhosis and iron storage grade 2 and had earlier suffered from heavy alcohol intake. Two other patients had microscopic sign of iron overload (grade 2), without any other obvious pathology. However the amount and distribution was not typical for hemochromatosis (not hepatocytic).

Sixteen patients were offered phlebotomy due to symptoms described earlier. Mean volume of whole blood needed for reaching low normal ferritin level (<100 µg/l) was 2500 ml (1300 ml–4450 ml). Accordingly approximately 1150 mg iron was removed for reduction of s-ferritin to low normal levels.

**Figure 1:**
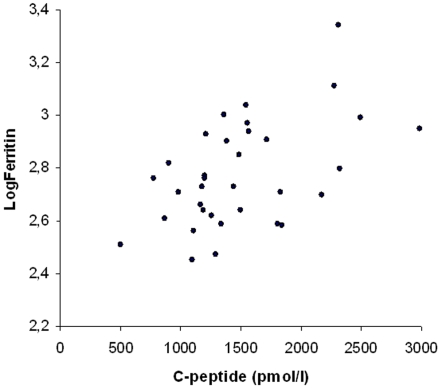
Relation between logferritin and C-peptide.

**Figure 2:**
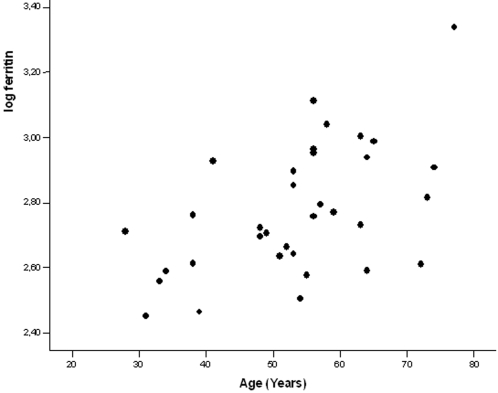
Relation between logferritin and age.

In univariate linear regression analysis log s-ferritin was significantly related to age (p<0.002) and insulin C-peptide (p<0.002). These parameters were also significantly related to s-ferritin in multivariate regression analysis (p<0.002) (Fig. 1 and 2). There was no significant relation between s-ferritin and transferrin saturation, number of phlebotomies required to normalize s-ferritin, ALT, LDL-cholesterol, HDL-cholesterol, trigyserides, diastolic blood pressure or BMI.

Eighteen of thirty-four (52%) patients fulfilled the IDF-criteria. In the 18 men fulfilling the criteria relative to the other male patients median ferritin was 603 µg/l (379–1300) compared to 515 µg/l (283–871) (p = 0.05), transferrin saturation was 36% compared to 44% (p<0.05) and mean insulin C-peptide was 1732 pmol/l compared to 1162 pmol/l (p = 0.001).

All liver biopsies showed steatosis in patients fulfilling the IDF-criteria, with only one showing an iron content corresponding to grade 2, the rest grade 0–1 (normal).

## Discussion

Hepatic iron overload was present in only 4 patients (4/29). These had also slightly elevated transferrin saturation. In two of them increased iron stores were explained by alcohol consumption. Liver histology, transferrin saturation, and number of phlebotomies needed to normalize s-ferritin, indicated normal iron-load in the remaining population. There was no sign of inflammation or malignancy. Steatohepatitis was found in three patients. One of these was diagnosed as NASH.

The majority of the study population showed markers of the metabolic syndrome. BMI, waist-hip ratio, diastolic blood pressure, ALT, HDL-cholesterol, fasting plasma glucose and insulin C-peptide showed deviations typically seen in the metabolic syndrome. Elevated insulin C-peptide levels indicate hyperinsulinemia and insulin resistance [20]–[21]. Liver histology showed steatosis and nuclear glycogen inclusions, a typical finding in prediabetes and DM2.

Those patients who met the criteria for the metabolic syndrome had slightly higher ferritin and C-peptide values, but lower transferrin saturation than the rest of the study population. One of these had iron storage grade 2, the rest grade 0–1 in liver biopsies.

An association between hyperferritinemia and insulin resistance in patients with different types of liver pathology has been reported earlier. In the article by Moirand et al [5] the patients had overt hepatic iron overload, as the median liver iron concentration was 85 µmol/g dry weight (normal <36 µmol/g).

A relationship between non-alcoholic fatty liver disease and insulin resistance [8], and a relationship between s-ferritin and metabolic markers of insulin resistance but without an evaluation of liver-histology [22]–[23], has previously been reported. In 1992 Dinnen et al reported that hyperferritinemia was a feature of newly diagnosed DM2 [24], and they later presented results indicating that DM2 did not associate with iron overload [25]. This finding has recently been confirmed by others [26]. Inflammatory mechanisms were assumed to be responsible for the hyperferritinemia in these diabetic patients.

Our patients differed from those in the above mentioned studies by being mostly non-diabetic, without hepatic iron overload or other sign of liver pathology other than steatosis and nuclear glycogen inclusions. Our patients had more expressed markers of the metabolic syndrome than in the above studies, and mean ferritin levels were higher. This tendency was even more striking in the group with the metabolic syndrome. In addition ferritin levels correlated well with fasting insulin C-peptide.

Elevated ferritin levels in our patients seemed not to be due to iron. As elevated insulin C- peptide level is a marker of hyperinsulinemia and indirectly insulin resistance, insulin resistance might be the cause of elevated ferritin levels in our patients, either directly or due to the steatosis itself.

Patients treated with phlebotomy experienced reduction in s-ferritin, but after a while the level gradually increased. Those who changed lifestyle (weight management, increased physical activity) seemed to experience more stable s-ferritin reduction. This has to be evaluated in further studies, and in particular correlation needs to be observed between a durable fall in serum ferritin in response to weight control and exercise, and a correction in insulin resistance and reversal of fatty liver.

The finding of steatosis and nuclear glycogen inclusions further support this theory, as it is a common finding in obesity and prediabetes. We did not find signs of primary liver disease.

The frequency of hyperferritinemia not related to iron load is remarkable, as is the extreme male preponderance. Insulin resistance and metabolic syndrome is more prevalent in males [18], but not to the extent seen in this group. Confounding factors may have influenced patient selection, but hormonal effects and increased iron loss in females may also have been of importance. Selection bias on part of the referring physician is not possible to exclude.

Possible explanations of hyperferritinemia among our patients might be that by binding iron, ferritin protects against formation of free hydroxyl groups, it could also be due to endothelial/subendothelial inflammation [27]–[28] or simply leakage of ferritin from hepatic cells.

The frequency of hyperferritinemia in Norwegian unselected patient populations with insulin resistance is unknown, as are gender difference.

The metabolic syndrome carries a well known increased risk for cardiovascular disease [29]–[30]. Whether hyperferritinemia entails an additional risk is unknown.

The greatest limitation of the study is the relatively small patient population, and male preponderance. The strength though, is the unselected patient population from a primary care hospital, and the performance of liver histology from most of the patients.

In conclusion, the present study suggests that s-ferritin elevation in our patients is a marker of the metabolic syndrome with hepatic steatosis and insulin resistance, and not of iron overload. The direct pathogenic mechanism, however, remains unknown.
